# Polydatin Reverses Diabetes‐Induced Apoptosis of Pancreatic *β*‐Cells by Inhibiting the TNF/TNF‐R1/Caspase‐3 Signaling Axis

**DOI:** 10.1155/jdr/9073132

**Published:** 2026-09-12

**Authors:** Jun Li, Yifan Zhou, Xuetong Wu, Guo Cheng, Qun Ma, Yixue Zhou, Nannan Zhang

**Affiliations:** ^1^ School of Basic Chinese Medicines, Guizhou University of Traditional Chinese Medicine, Guiyang, China, gzu.edu.cn; ^2^ School of Pharmaceutical Sciences, Guizhou University of Traditional Chinese Medicine, Guiyang, China, gzu.edu.cn; ^3^ School of First Clinical Medical, Guizhou University of Traditional Chinese Medicine, Guiyang, China, gzu.edu.cn; ^4^ School of Basic Medicine, Guizhou University of Traditional Chinese Medicine, Guiyang, China, gzu.edu.cn

## Abstract

**Background:**

Pancreatic *β*‐apoptosis is a critical pathological event in diabetes mellitus (DM), yet effective therapeutic agents that directly protect *β*‐cells are lacking. Polydatin (PD), a natural stilbenoid, has shown potential in regulating glucolipid metabolism; however, its role and mechanism in protecting pancreatic *β*‐cells from apoptosis remain largely unexplored.

**Objective:**

The present work focused on investigating whether PD prevents pancreatic *β*‐cells against diabetes‐related apoptosis and exploring the associated molecular mechanisms, with a particular focus on the TNF/TNF‐R1/caspase‐3 signaling axis.

**Methods:**

Network pharmacology and bioinformatics were applied in combination with experimental validation. Potential targets of PD and DM were retrieved from public databases. Key candidate targets were screened via protein–protein interaction (PPI) analysis, least absolute shrinkage and selection operator (LASSO) regression, functional enrichment (GO/KEGG), differential expression, receiver operating characteristic (ROC), and clinical correlation analyses. Binding interactions were assessed by molecular docking alongside dynamic simulations. In vitro and in vivo DM models were established with high glucose (HG)–stimulated MIN6 *β*‐cells and high‐fat/HG diet‐fed mice with low‐dose streptozotocin (STZ) administration, respectively.

**Results:**

TNF was identified as a central target of PD against DM, with elevated expression under diabetic conditions and a significant correlation with fasting blood glucose levels. PD exhibited strong binding affinity to both TNF (Vina score: –8.9 kcal·mol^−1^) and TNF‐R1 (Vina score: –8.8 kcal·mol^−1^). Cellular experiments demonstrated that PD enhanced *β*‐cell cycle progression (upregulated Ki67 expression), downregulated TNF‐R1 expression, and facilitated insulin secretion under HG stimulation. In diabetic mice, PD significantly increased the islet *β*‐cell mass and area; downregulated the expression of TNF, TNF‐R1, and cleaved‐caspase‐3; and restored PARP1 expression.

**Conclusion:**

This study revealed that PD attenuated diabetic *β*‐apoptosis, likely through inhibition of the TNF/TNF‐R1/caspase‐3 pathway, suggesting that PD may be a novel anti‐DM therapeutic target.

## 1. Introduction

Diabetes mellitus (DM), a chronic metabolic disorder, is characterized by hyperglycemia caused by impaired insulin release and/or activity [1]. Among the various pathological processes underlying DM, pancreatic *β*‐apoptosis represents a critical event that drives the progressive decline in insulin production and exacerbates glycemic dysregulation. In Type 1 and Type 2 DM, *β*‐cell mass is lost, which ultimately affects disease progression and treatment outcomes [2, 3]. Despite substantial advances in glucose‐lowering pharmacotherapy, current strategies remain largely symptomatic and have limited efficacy in directly preserving *β*‐cell survival and function. This unmet clinical need has prompted increasing interest in identifying novel therapeutic agents that can protect *β*‐cells from apoptosis.

Natural products derived from medicinal plants are promising sources of bioactive components with potential antidiabetic activities. Among these, *Polygonum cuspidatum* has drawn considerable attention because of its documented hypoglycemic and lipid‐lowering activities [4, 5]. PD (PD; 3,4,5‐trihydroxystilbene‐3‐*β*‐D‐glucoside), a major stilbenoid constituent separated from the roots and rhizomes of *P. cuspidatum*, serves as the natural resveratrol precursor, and its concentrations are substantially higher than those of resveratrol in this plant [6, 7]. Notably, compared with resveratrol, PD has superior oral bioavailability and metabolic stability, making it a more pharmacologically attractive candidate [8]. Accumulating evidence has demonstrated that PD has pleiotropic biological activities, such as anti‐inflammatory, antiapoptotic, glucolipid regulatory, and antioxidant effects [9–14]. However, despite these promising properties, the precise role of PD in protecting pancreatic *β*‐cells against diabetes‐induced apoptosis and its associated molecular mechanisms is unclear.

Recent advances in network pharmacology and bioinformatics have enabled systematic prediction of drug‐target interactions, offering a powerful approach to uncover novel mechanisms of action for natural compounds. In this work, network pharmacology, bioinformatics analysis, and both in vitro and in vivo experimental validation were performed to explore whether PD can protect pancreatic *β*‐cells against diabetes‐related apoptosis and elucidate the key signaling pathways involved. Our findings support the idea that PD may be a *β*–cell‐protective agent for treating DM.

## 2. Materials and Methods

### 2.1. Animal Model Establishment

PD was purchased from MedChemExpress (No. 27208‐80‐6). Forty 4–5‐week‐old C57BL/6 male mice (SPF grade) weighing 17–20 g were obtained from Changsha Tianqin Biotechnology Co. Ltd. (certificate of conformity No. SCXK (Xiang) 2022–0011). After 5 days of adaptation to standard chow, 10 of them were randomized into the control group to be fed standard chow, whereas the remaining animals were given high‐fat chow (60% fat diet, 12492 M, from Beijing Boaigang Biotechnology Co. Ltd). The specific feeding method followed was to overfeed half of the high‐fat chow and half of the standard chow on the 6th–7th day, and the high‐fat chow was started on the 2nd week and continued. High‐fat chow feeding was started and continued for 4 weeks until the body weight reached more than 20–25 g. These mice were used for streptozotocin (STZ)‐induced modeling. STZ (60 mg·kg^−1^; B2001; from Beijing Boaigang Biotechnology Co. Ltd.) was injected intraperitoneally for three consecutive days. Fasting blood glucose levels (mice fasted for 6–8 h) were measured with a blood glucose meter (Sinocare Biosensing Co. Ltd.) on Days 3, 7, and 10 by collecting mouse tail venous blood, and a concentration of > 11.1 mM was considered successful. Successfully modeled mice were fed high‐fat chow and randomized into four groups (*n* = 10 each): control, model, PD low‐dose (100 mg·kg^−1^ via gavage), and PD high‐dose (200 mg·kg^−1^ by gavage) groups. Animals from diverse groups were given the respective drug solution or saline at 6 PM through gavage daily for 8 weeks. Two hours after the last dose was administered, under the intraperitoneal anesthesia of 1% pentobarbital sodium injection (0.005 mL·g^−1^), eyeball blood samples were harvested, followed by standing for a 1‐h duration under ambient temperature. Blood samples were centrifuged at 3000 r/min (r = 8 cm) and 4°C for 15 min, after which the upper yellowish serum layer was collected for preservation at −20°C. A portion of pancreatic tissue was immersed in 4% paraformaldehyde solution, whereas the other part was stored in liquid nitrogen. All experiments were performed in accordance with the relevant Chinese laws, Chinese animal ethical guidelines, and experimental protocols. Our animal experiments were approved by the Experimental Animal Ethics Review Committee of Guizhou University of Traditional Chinese Medicine (No. 20230224).

### 2.2. OGTT Glucose Tolerance Test

In Week 8, the mice in the control, model, and PD groups were fasted but supplied with normal water for 16 h and then orally gavaged with D‐glucose at 2 g·kg^−1^ body weight. Blood glucose levels were measured at 0 min prior to glucose consumption and at 30, 60, and 120 min following glucose consumption. Mouse‐tail venous blood was harvested.

### 2.3. Lipid Quadruple Test

During the 8th week after modeling, the mice were fasted but normally supplied with water for 16 h. Whole blood was collected from the eyeballs and left for a 1‐h period at ambient temperature, followed by 15 min of centrifugation at 3000 r/min (*r* = 8 cm) and 4°C to collect the upper yellowish serum layer for the detection of four indices of lipids, namely, total cholesterol (TC), triacylglycerol (TG), high‐density lipoprotein cholesterol (HDL‐C), and low‐density lipoprotein cholesterol (LDL‐C).

### 2.4. Hematoxylin and Eosin (HE) Staining

The 4% neutral formaldehyde‐fixed pancreatic tissue was dehydrated, after which it was dipped in wax and embedded in paraffin to prepare wax blocks. The 4‐*μ*m thin paraffin‐embedded tissue sections were cut using a semiautomatic slicer and subjected to HE staining. After the sections were deparaffinized with xylene, they were hydrated with a descending gradient of ethanol. Afterward, pancreatic tissue sections were prepared for HE staining following HE staining kit protocols (Beijing Solarbio Science & Technology Co. Ltd.), and the sections were scanned using a section scanner. The obtained results were processed using CellSens software.

### 2.5. High‐Glucose (HG) Cell Model Establishment

Mouse pancreatic islet *β*‐cells (Min6) were obtained from Xavier Biobank (No. STCC20029P‐1) and cultured in Gibco 1640 medium supplemented with 10% fetal bovine serum (FBS) and a 1% penicillin–streptomycin mixture at 37°C and 5% CO_2_. These cultured cells were classified into the control, HG, and PD treatment groups. Afterward, the cells were digested when they were 80% confluent. Following a 24‐h culture process, cell interactions were performed with PD at 10, 20, and 40 *μ*M. The HG concentration was chosen to mimic the HG microenvironment because it did not differ from that of the control group in terms of insulin secretion and significantly decreased the cell proliferation rate.

### 2.6. Cell Proliferation Assay

Min6 cells were cultivated, and the cell density was adjusted such that 5 × 10^3^ cells/well were added to a 96‐well plate. When the cells reached 80% confluence, the supernatant was discarded, and the cells were subsequently washed once with PBS. Subsequently, 200 *μ*L/well of 25 mmol·kg^−1^ HG and varying concentrations of PD (10, 20, and 40 *μ*M) were added to each group for a 24‐h incubation duration. Afterward, 20 *μ*L of MTS reagent was added to each well, and the samples were incubated for 1 h. Later, the absorbance (OD) values were determined using an enzyme marker. The equation below was used to determine the cell proliferation rate:

Percent cell proliferation rate=absorbance in experimental group−absorbance in blank controlabsorbance in control group−absorbance in blank control×100%.

### 2.7. Effects of PD on Mouse Pancreatic *β*‐Cells and Mouse Serum Insulin Secretion

The density of the Min6 cells in the five groups was adjusted to 5 × 10^5^ cells/well before they were inoculated in 6‐well plates. The cells were spread over 80% of the bottom of the wells, and then the supernatant was discarded, followed by washing with PBS once. To each well, 200 *μ*L of 25 mmol·kg^−1^ HG and varying concentrations of PDs (10, 20, 40 *μ*M) were introduced into every group before 24 h of incubation. Afterward, the cell supernatants were removed.

Blood supernatants were collected from the control, model, low‐dose (100 mmol·kg^−1^), and high‐dose (200 mg·kg^−1^) PD groups. The ELISA kit (JingMei Biotechnology, Item No. JM‐02862M2,48T) was subjected to 60 min of equilibration under ambient temperature from the refrigerator at 4°C. Horseradish peroxidase (HRP)‐conjugated antibody (100 *μ*L) was added to every well. Later, these wells were sealed using a plate sealing membrane for 60 min of incubation at 37°C. After the plate was washed, 50 *μ*L each of substrates A and B was added to every well for an additional 15 min of incubation at 37°C. The termination solution (50 *μ*L) was introduced into every well, and the OD values were measured at 450 nm for 15 min. The sample concentration was measured in line with the calibration curve equation.

### 2.8. Protein Immunoblotting

Min6 cells and pancreatic tissues were harvested for protein immunoblotting (western blot, WB) analysis. After quantification using the BCA Protein Detection Kit (Solarbio, Item No. PC0020), protein aliquots were separated by SDS–PAGE (Solarbio, Item No. P1200) prior to their transfer to a PVDF membrane, which was subsequently blocked by 2 h of incubation with 5% skim milk. The antitumor necrosis factor (TNF) antibody (Affinity Bioscience. AF7014#29, 1:500), a TNF‐R1 antibody (Affinity Bioscience, AF0282##29, 1: 1000), and a rabbit anti‐Ki‐67 antibody (Bioss Antibodies, bs‐23103R, 1: 1000), followed by incubation with goat antirabbit IgG (H&L)‐HRP (Bioworld, #bs13278, 1: 8000) for 2 h. An ECL kit (Millipore, Batch No. 2201215) was used for development. Finally, Image Lab software was used to analyze the band grayscale values, and the target protein‐to‐*β*‐actin grayscale value ratio was used for semiquantitative analysis.

### 2.9. Immunohistochemistry

The pancreatic paraffin sections were degummed by immunohistochemistry, the antigen was repaired with citrate buffer, and 3% hydrogen peroxide was added to block endogenous peroxidase activity, followed by 15 min of incubation at ambient temperature. Primary antibodies, including anticaspase‐3 (1:1000; Wuhan Saierwei Technology Co. Ltd.; GB155600), cleaved‐caspase‐3 (1:1000; Wuhan Saierwei Technology Co. Ltd.; GB11767C), TNF (1:1000; Wuhan Saierwei Technology Co. Ltd.; GB153968), and PARP1 (1:1000; Wuhan Saierwei Technology Co. Ltd.; GB111500), were subsequently added and incubated at 4°C overnight. HRP‐conjugated secondary antibodies were subsequently added for another 1 h of incubation at ambient temperature prior to HE staining. The relative expression rates of the caspase‐3‐, cleaved‐caspase‐3‐, and TNF‐positive regions were observed and calculated by light microscopy.

### 2.10. Common Target Prediction of PD and DM

PD structure (SMILE format) was obtained through PubChem, and its targets were acquired from Traditional Chinese Medicine Systems Pharmacology (TCMSP), the Super‐Enhancer Archive (SEA), PharmMapper, SuperPred, the Comparative Toxicogenomics Database (CTD), and SwissTargetPrediction (excluding targets with likelihood < 0). Further, DM targets were identified via the DisGeNET database. Potential overlapping targets between PD and DM were obtained by intersecting drug and disease targets via Venny2.1 (https://bioinfogp.cnb.csic.es/tools/venny/).

### 2.11. Protein–Protein Interaction (PPI) Network Establishment and Pivotal Target Selection

A PPI analysis was conducted on the predicted potential common targets with STRING database (https://cn.string-db.org) at the 0.4 confidence level. The results from the analysis were input into Cytoscape 3.7.2 software and visualized with a combined score to obtain protein–target interaction maps. The intersecting targets were screened by the Matthews correlation coefficient (MCC) algorithm through the CytoHubba plug‐in to select the Top 10 hub targets.

### 2.12. Establishment of Least Absolute Shrinkage and Selection Operator (LASSO) Models

Ten common targets were further filtered using the dimensionality reduction model LASSO regression. LASSO performs superior regression analysis in assessing high‐dimensional results while using the regularization method to improve prediction performance. LASSO analysis with 11‐fold cross‐validation was performed with the “glmnet” R package. The parameters are fixed below: lasso. *n* = 11, nfolds = 7, split_train = 0.4. The genes were selected as common targets by rotating or penalizing the parameters, and nonzero genes were considered common targets, whereas filtered genes were key intersection targets.

### 2.13. GO and KEGG Analyses of Key Targets

GO and KEGG analyses for common targets were conducted with the KEGG REST API (https://www.kegg.jp/kegg/rest/keggapi.html) [15–17] and DAVID databases. These annotations were used as backgrounds for mapping genes into the background set, and the R clusterProfiler package (Version 3.14.3) was then adopted for conducting gene set enrichment analysis, with analysis parameters including a minimum gene number of 5 and a maximum gene number of 5000. *p* < 0.05 indicated statistical significance.

### 2.14. Validation of Key Target Differential Expression and Receiver Operating Characteristic (ROC) Analysis

Two external datasets related to DM (GSE156993 and GSE78721) were used, with *p* < 0.05 and |log2*F* *C*| > 1 as the screening conditions. The independent Wilcoxon rank sum test method was adopted to verify key intersection target levels in the DM group versus the normal group. The diagnostic significance of the intersecting differential targets was evaluated using the “pROC” R package, which created ROC curves that illustrate the relationship between sensitivity and specificity. The X‐coordinate represents 1‐specificity (false‐positive rate), with the accuracy as it is closer to zero. Moreover, the Y‐coordinate denotes sensitivity (true positive rate), with higher values indicating accuracy. The area under the curve (AUC), which is 0.5–1, is often used to evaluate diagnostic tests, with an AUC value approaching 1 indicating the superior diagnostic accuracy of the variable in predicting the outcome.

### 2.15. Clinical Correlation Analysis

Clinical data on fasting blood glucose levels for key targets were analyzed based on the Nephroseq database (https://nephroseq.org), and the clinical relevance of the intersecting targets was verified.

### 2.16. Molecular Docking

The binding affinity between common targets and major components of PD was calculated by CB‐Dock2 (https://cadd.labshare.cn/cb-dock2/php/index.php), the molecular docking tool that employs AutoDock Vina calculations. It is adopted for identifying ligand–receptor binding regions and examining their center position and size. Protein structure data were retrieved from the UniProt (https://www.uniprot.org/) and STRING databases, and 3D structures were acquired from the RCSB PDB protein database (https://www.rcsb.org/). The PubChem database (https://pubchem.ncbi.nlm.nih.gov/) was adopted for acquiring the small‐molecule structure (in.sdf format) of the PD‐active compound, which was further processed by adding hydrogen atoms, removing all water molecules, and minimizing free energy. During this analysis, it was ensured that the entire molecule of each component was contained within the active center. The best docking complex was selected based on the binding energy score predicted by CB‐Dock2 and Vina, with smaller Vina scores representing better binding. The docking results were represented under the following conditions: (1) the ligand and receptor docking results were set to “Cartoon” mode; (2) for ligands and colored receptors, the “Ligand” and “Secondary structure” settings were used.

### 2.17. Molecular Dynamics Simulation

Molecular dynamics simulations can be used to elucidate the binding affinity of small molecules within a binding site and investigate complex stability. In this study, MDS analysis was carried out for 100 ns to simulate the complex labeled “2E7A/PD” using the “Desmond” module. An explicit solvent model, including the TIP3P water molecule, was used, and the intermolecular interactions were represented by the OPLS‐2005 force field. To guarantee that this system was accurately represented, simulations were performed inside a periodic bounding salver box (100 × 100 × 100 Å in size). To achieve a charge balance, we added 0.15 M sodium ions (Na^+^) to this system. Further, the addition of a sodium chloride solution helps mimic physiological conditions, thus generating a similar simulated biological environment. The simulation of the whole system (encompassing complexes, solvents, and ions) was performed for 100 ns, thus shedding more light on the “2E7A/PD” complex′s dynamic behavior in this environment.

### 2.18. Statistical Analysis

The data were visualized using GraphPad Prism 9. When multiple comparisons were being conducted, one‐way ANOVA was adopted for normally distributed data with homogeneity of variance. When two groups are compared, if the data satisfy normality, the independent sample *t* test is used. *p* < 0.05 suggested statistical significance.

## 3. Results

### 3.1. Identification of Key Targets for PD Treatment in Diabetes

**Figure 1:**
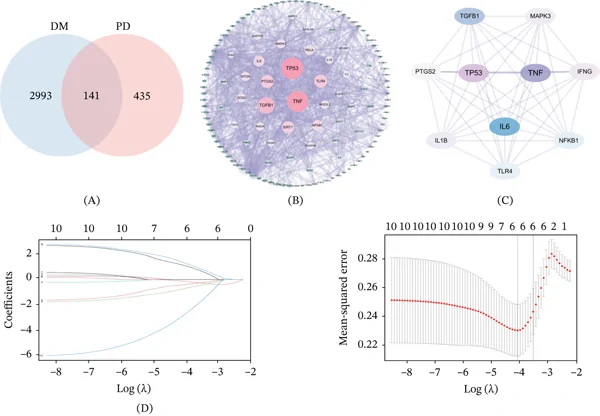
Identification of key targets for PD treatment in diabetes. (A) The intersection diagram of PD and DM, (B) PPI network analysis on intersected targets, (C) the Top 10 targets, and (D) six key targets were screened by LASSO modeling.

A total of 576 PD targets were obtained from TCMSP, PharmMapper, SuperPred, SEA, CTD, and SwissTargetPrediction. Further, 1189 diabetes targets were identified by the DisGENT database. Overall, 141 common targets for PD and DM were obtained by intersecting disease and drug targets (Figure 1A). These common targets were subsequently input into the STRING database for PPI analysis. Afterward, the resulting PPI analysis results were imported into Cytoscape 3.7.2 software for visualization, and finally, an interaction map was constructed between PDs and DM common protein targets (Figure 1B). The Top 10 targets, namely, TP53 (tumor protein P53), IL6 (interleukin 6), IL1B (interleukin 1 beta), TNF, TGF*β*1 (transforming growth factor beta 1), PTGS2 (prostaglandin‐endoperoxide synthase 2), MAPK3 (mitogen‐activated protein kinase 3), TLR4 (toll like receptor 4), NF*κ*B1 (nuclear factor kappa B subunit 1), and IFNG (interferon gamma), were screened based on the MCC algorithm (Figure 1C). LASSO analysis with 11‐fold cross‐validation by rotation or penalty parameters identified six nonzero genes as key targets (Figure 1D), namely, TP53, IL6, TNF, TLR4, NF*κ*B1, and IFNG.

### 3.2. GO and KEGG Analyses of Six Key Targets

**Figure 2:**
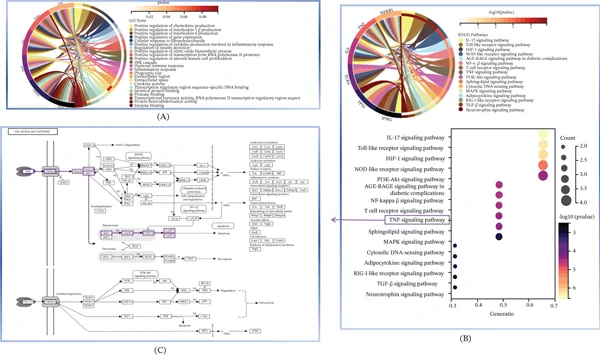
GO and KEGG analyses for six key targets. (A) GO annotation, (B) KEGG analysis, and (C) TNF signaling pathway map.

GO enrichment analysis revealed that the six intersecting targets (TP53, IL6, TNF, TLR4, NF*κ*B1, and IFNG) are primarily associated with the positive regulation of chemokine, interleukin‐1*β*, and IL6 production. Additionally, these targets are related to the positive regulation of gene expression, the cellular response to lipopolysaccharide, and the inflammatory response (Figure 2A). Further, according to the results of the KEGG analysis, the six intersecting targets were mostly related to the IL‐17, TNF, MAPK, HIF‐1, toll‐like receptor, and NF‐*κ*B signaling pathways, along with other DM‐related pathogenic processes (Figure 2B). Moreover, GO and KEGG analyses suggested that these biological functions and pathways were closely related to the pathogenesis of DM.

### 3.3. Validation of Differential Expression and ROC Analysis of Six Key Targets

**Figure 3:**
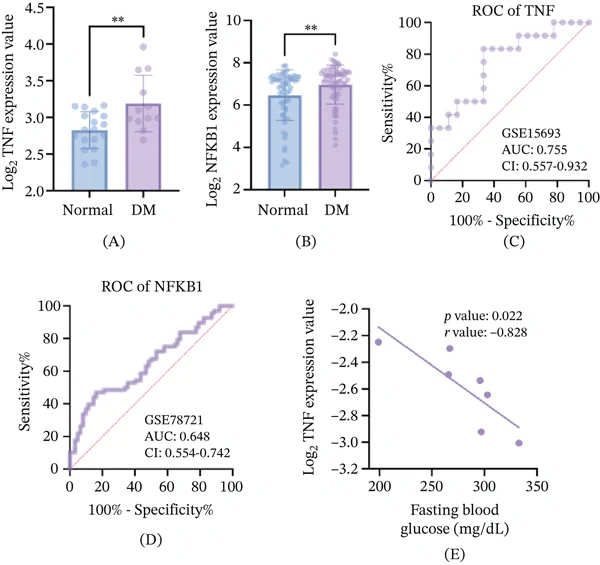
Differential expression, ROC, and clinical relevance analysis of key targets. (A) Differential expression analysis, (B) ROC analysis, and (C) clinical relevance analysis, ∗∗*p* < 0.01 versus normal group.

To verify the differential expression levels of key targets (TP53, IL6, TNF, TLR4, NF*κ*B1, and IFNG) in DM and their diagnostic value, two DM‐related validation sets (GSE156993 and GSE78721) were used. Compared with those in normal tissue, TNF levels markedly increased in the DM group of the GSE156993 dataset. Compared with that in normal tissue, the expression of NF*κ*B1 markedly increased in the DM group in the GSE78721 dataset (Figure 3A).

We subsequently assessed the classification ability of TNF and NF*κ*B1 for the DM and normal groups through ROC curve analysis. The ROC curve results revealed that TNF had an AUC value of 0.755 in the GSE156993 dataset, whereas NF*κ*B1 had an AUC value of 0.648 in the GSE78721 dataset (Figure 3B). An AUC value approaching 1 indicates superior classification ability. For TNF, the sensitivity, specificity, and accuracy of the model were 83.33%, 66.67%, and 75%, respectively, at the optimal threshold of 2.940, whereas those for NF*κ*B1 were 47.06%, 83.87%, and 65.44%, respectively, at the optimal threshold of 7.366. These findings indicate that TNF is more accurate and reliable for identifying patients with diabetes.

### 3.4. Clinical Correlation Analysis

Clinical TNF fasting blood glucose data were extracted from the Nephroseq database and are presented as the mean ± standard deviation (SD). *p* < 0.05 suggested statistical significance of the variability. GraphPad Prism 7.0 software was subsequently used for linear regression, which revealed that TNF levels were significantly correlated with fasting blood glucose levels (Figure 3C).

### 3.5. Molecular Docking

**Figure 4:**
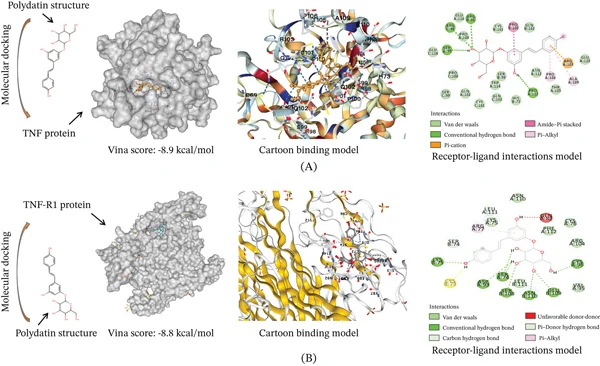
Molecular docking analysis of polydatin with TNF and TNF‐R1. (A) Optimal conformation of TNF with polydatin. (B) Optimal conformation of TNF‐R1 with polydatin.

The protein structures of TNF (PDB ID: 2e7a) and TNF‐R1 (PDB ID: 1ext) and the PD structure (CID: 5281718) were subjected to CB‐dock2 for molecular docking simulation analysis, and the docked conformations that had the lowest binding energy values were chosen (Table 1). The ability of the ligand to bind to the receptor was evaluated based on the Vina score, with a smaller Vina score indicating more stable and efficient ligand–receptor binding affinity. The best docking conformation of TNF with PD was located at binding pocket C2, and the Vina score was –8.9 kcal·mol^−1^ (Figure 4A). The optimal docking conformation for TNF‐R1 and PD was found at binding pocket C1, and the Vina score was –8.8 kcal·mol^−1^ (Figure 4B).

**Table 1:** The binding energy of TNF and receptor TNF‐R1 with PD.

Protein	Vina score (kcal·mol^−1^)	Cavity volume (Å^3^)	Center (*x*, *y*, *z*)	Docking size (*x*, *y*, *z*)
TNF	−8.9	1038	−2, −7, 3	23, 23, 23
TNF‐R1	−8.8	2759	0, 36, −6	33, 23, 32

### 3.6. Molecular Dynamics Simulation

**Figure 5:**
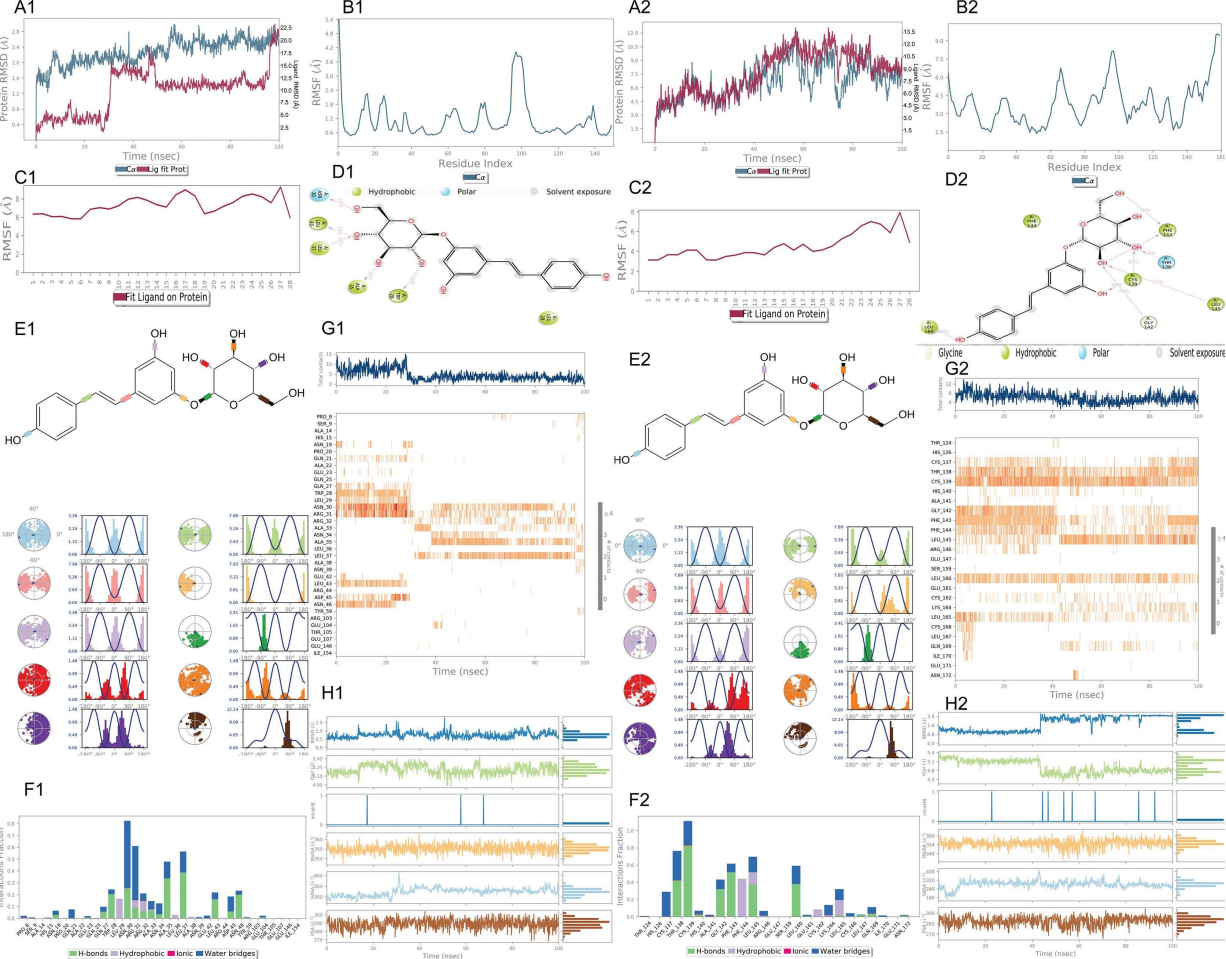
Molecular dynamics simulation analysis of the system of complex TNF‐PD and TNF‐R1‐PD. (A1,A2) RMSD plot, (B1,B2) RMSF analysis, (C1,C2) ligand RMSF, (D1,D2) ligand‐protein docking plot, (E1,E2) ligand torsion profile plot, (F1, F2) protein–ligand interaction plot, (G1,G2) timeline of amino acid residue interactions over 100 ns of simulation, and (H1,H2) ligand properties of compound PD.

As revealed by MD analysis, the proteins exhibited significant conformational changes during the simulation, with a stable fixed value of root mean square deviation (RMSD) (Figure 5A1,A2). Furthermore, the peak value of protein RMSF represents the protein region with the greatest fluctuation during the simulation process (Figure 5B1,B2). The RMSF fluctuation of the protein is relatively small, and thus, it performs stably during the simulation process. The ligand RMSF shows the ligand fluctuations decomposed by atoms (Figure 5C1,C2), and the results indicate that the fluctuations were small during the simulation process, suggesting that the simulation process was stable. Protein molecular interaction plots (Figure 5D1, E1, and F1) depict the hydrophobic interactions of PD ligands in the TNF‐PD complex with the amino acid residues LEU29, ARG31, ARG32, ALA33, ALA36, and ALA38. Protein molecular interaction plots (Figure 5D2, E2, and F2) depict the hydrophobic interactions of PD ligands in the TNF‐R1‐PD complex with the amino acid residues LEU160, LEU145, PHE144, PHE143, CYS139, and THR138. Furthermore, protein–ligand interactions were observed throughout the simulation, and some amino acid residues exhibited more than one specific type of contact with the ligand, which is indicated by a darker orange color. A timeline for amino acid residue interactions during 100 ns of simulation is shown in Figure 5G1,G2, offering reliable information for understanding molecular‐level complex dynamic behavior. The “ligand–protein” contact map is shown in Figure 5H1,H2, underscoring that the interactions lasted more than 5.0% of the time in those chosen trajectories (0.00–100.00 ns), and key amino acid residues were located at the core of these interactions.

### 3.7. Role of PD in Mouse Blood Glucose

The results of the mouse oral glucose tolerance test (OGTT) at 0, 30, 60, 90, and 120 min revealed that the model group and the high and low doses of PD impaired glucose tolerance in the treated group of mice (File S1).

### 3.8. Effects of PD on the Four Lipid Profiles in Mice

The reference range of TG concentration was 0–1.7 mmol·kg^−1^; that of TC (CHOL) ranged from 3–5.2 mmol·kg^−1^; that of HDL‐C ranged from 0.9–2 mmol·kg^−1^; and that of LDL‐C ranged from 0–3.1 mmol·kg^−1^. The results of the serum lipid profile in the mice revealed that PD did not significantly decrease the lipid profile (File S2).

### 3.9. PD Promotes Pancreatic Islet Cell Recovery in Diabetic Mice

**Figure 6:**
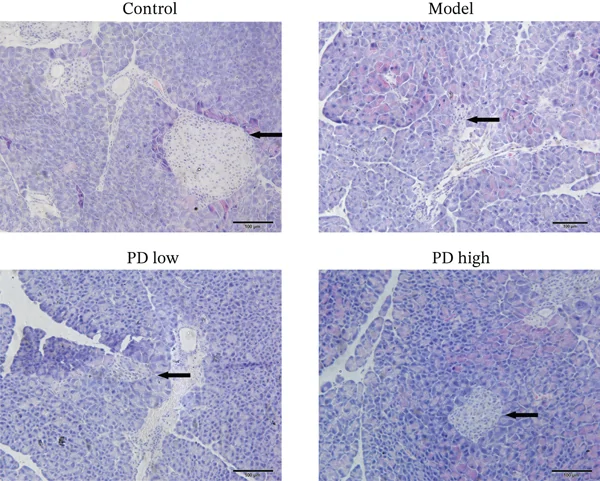
Histopathological sections of pancreatic tissue after PD intervention.

The histological results of C57BL/6 mice revealed a reduction in pancreatic islet quantity and area in both model and PD‐treated mice, along with border changes and vacuolization. However, the pancreatic islet quantity and area were markedly greater in the high‐dose PD‐treated group than in the model group (Figure 6).

### 3.10. PD Promotes Insulin Secretion in Min6 Cells and Diabetic Mice

**Figure 7:**
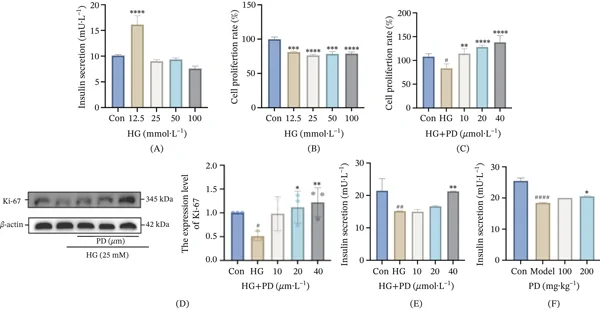
Insulin secretion after PD intervention. (A,B) Screening of high glucose modeling concentration (*n* = 5), (C) proliferation rate of Min6 cells after PD intervention (*n* = 5), (D) protein expression levels of Ki‐67 within Min6 cells (*n* = 3), (E) insulin production of Min6 cells after PD intervention (*n* = 3), and (F) insulin secretion in mice after PD treatment (*n* = 3), #*p* < 0.05, ##*p* < 0.01, and ####*p* < 0.0001 versus control group, ∗*p* < 0.05, ∗∗*p* < 0.01, ∗∗∗*p* < 0.001, and ∗∗∗∗*p* < 0.0001 versus model group or HG group.

First, we determined the appropriate HG concentration for Min6 cells using MTS and insulin secretion experiments. The cell culture results, shown in Figure 7A,B, revealed that insulin secretion in Min6 cells decreased at a HG concentration (25 mM) and that cell proliferation was inhibited. Hence, we used 25 mM as the modeling concentration and administered PD. These findings indicated that PD reversed the inhibition of proliferation caused by HG concentrations (Figure 7C), increasing the expression of the cell proliferation marker Ki‐67 (Figure 7D), and promoting insulin secretion (Figure 7E). Furthermore, animal experiments revealed that high PD concentrations could reverse insulin secretion (Figure 7F).

### 3.11. PD Inhibits the TNF/TNF‐R1/Caspase‐3 Signaling Axis

**Figure 8:**
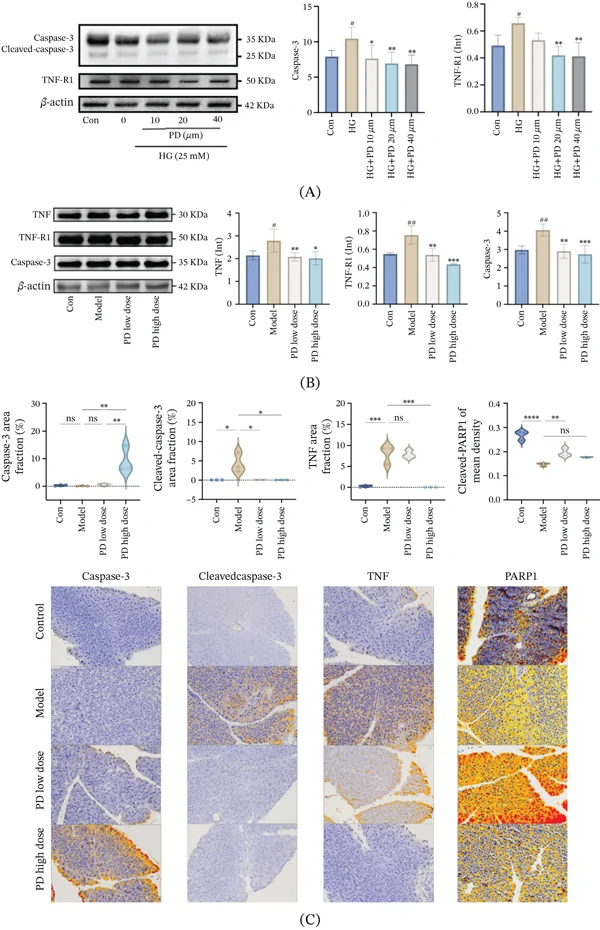
PD inhibits the TNF/caspase‐3 pathway. (A) WB of TNF‐R1 and caspase‐3 in diabetic Min6 cells (*n* = 3), (B) WB analysis of TNF, TNF‐R1 and caspase‐3 in diabetic pancreatic tissue (*n* = 3), (C) immunohistochemical analysis of caspase‐3, cleaved‐caspase3, TNF, and PARP1 in mouse pancreatic tissues (*n* = 3). #*p* < 0.05 and ##*p* < 0.01 versus control group; ∗*p* < 0.05, ∗∗*p* < 0.01, ∗∗∗*p* < 0.001, and ∗*p* < 0.0001 versus HG group.

Western blotting revealed that HG markedly increased TNF‐R1 and cleaved‐caspase‐3 levels in MIN6 cells, whereas PD treatment reversed these effects dose‐dependently (Figure 8A). Consistently, in pancreatic tissues from diabetic mice, the elevated expression of TNF, TNF‐R1, and cleaved‐caspase‐3 was markedly attenuated by PD administration (Figure 8B). Immunohistochemical staining further confirmed increased TNF and cleaved‐caspase‐3 levels in diabetic islets, accompanied by reduced total PARP1 expression, and PD administration effectively abolished these effects (Figure 8C). Collectively, these findings suggest that PD mitigates pancreatic *β*‐cell apoptosis through inhibition of the TNF/TNF‐R1/caspase‐3 signaling axis.

## 4. Discussion

DM is an intricate metabolic disease with a wide global impact [18, 19]. Its typical feature is chronic hyperglycemia caused by inadequate insulin levels or activity, which is associated with multiple organ dysfunctions and damage that ultimately leads to disease progression and complications [20]. Chronic hyperglycemia induces oxidative stress, resulting in impaired function and ultimately death of pancreatic *β*‐cells [21, 22]. High‐fat and high‐sugar diets induce insulin resistance [23], whereas STZ can impair pancreatic *β*‐cells while affecting insulin release, causing dysfunctional glucose disposal and elevated blood glucose levels [13]. Decreased insulin receptor kinase activity further worsens insulin sensitivity [24]. Moreover, insulin resistance leads to hyperglycemia, disrupting the ATP/ADP balance within *β*‐cells and impairing islet secretory function [25], creating a vicious cycle that exacerbates diabetic glucolipid metabolic disorders. Therefore, hyperglycemia has important effect on pancreatic *β*‐apoptosis and insulin release [2].

Through network pharmacology, we screened 141 potential targets of PD for treating DM, among which six key targets—TP53, IL6, TNF, TLR4, NF*κ*B1, and IFNG—were further identified using MCC and LASSO modeling algorithms. These targets were related mainly to the positive regulation of chemokine production and inflammatory responses. According to the results of the KEGG analysis, these targets were mostly linked to the IL‐17, TNF, and MAPK pathways, all of which are closely associated with DM pathogenesis. Differential expression, ROC, and clinical correlation analyses further revealed TNF as the most critical target. Molecular docking demonstrated stable and effective binding between TNF and PD, and the Vina score was –8.9 kcal/mol. These in silico predictions are consistent with the established role of TNF as a proinflammatory cytokine linked to *β*‐cell loss, impaired insulin release, and hyperglycemia in Type 1 DM pathogenesis [26–28].

Our in vitro experimental results revealed that PD promoted cell cycle progression in MIN6 *β*‐cells (as indicated by upregulated Ki67 expression) and enhanced insulin secretion under HG conditions. However, the OGTT in diabetic mice did not reveal a significant blood glucose‐lowering effect. This discrepancy may be attributed to insufficient insulin sensitivity or an insufficient amount of insulin secreted within a short timeframe to effectively reduce blood glucose levels (File S1)

Immunohistochemistry results demonstrated that PD significantly inhibited TNF and cleaved‐caspase‐3 expression in pancreatic islets while restoring total PARP1 expression. The proinflammatory factor TNF is widely associated with inflammatory responses and immune regulation. In diabetes, TNF overexpression is closely associated with pancreatic *β*‐cell dysfunction, as it exacerbates oxidative stress and apoptosis in islet cells by activating inflammatory pathways [26, 27]. Cleaved‐caspase‐3 is a key marker in the execution phase of apoptosis; its elevated expression indicates activation of the apoptotic signaling cascade. In the diabetic model group, the level of cleaved‐caspase‐3 was significantly increased, indicating that the apoptotic signaling pathway in islet cells was active. Conversely, in the PD‐treated groups, cleaved‐caspase‐3 expression was reduced, indicating that PD effectively blocked or inhibited the activation of apoptotic signaling and reduced islet apoptosis.

PARP1 is a key DNA repair enzyme that maintains genomic stability by repairing DNA damage under physiological conditions. However, during apoptosis, PARP1 is cleaved by caspase‐3, resulting in its inactivation and degradation [15, 16]. In the diabetic model group, total PARP1 expression was decreased, which is consistent with caspase‐mediated PARP1 cleavage and degradation during *β*‐apoptosis. Following PD treatment, PARP1 expression was substantially restored, which correlated with reduced cleaved caspase‐3 levels. These findings indicate that PD attenuated caspase‐mediated PARP1 degradation. Therefore, the observed changes in PARP1 expression serve as complementary evidence for reduced apoptosis, supporting our conclusion that PD inhibits the TNF/TNF‐R1/caspase‐3 signaling axis. Combining these results with molecular docking and MD simulation findings, PD may reverse diabetic islet *β*‐apoptosis by modulating the TNF/TNF‐R1/caspase‐3 pathway, thereby providing a promising anti‐DM treatment strategy.

Notably, the lipid profile test did not reveal that PD significantly decreased lipid levels in high‐fat diet (HFD)‐fed diabetic mice (File S2). These findings suggest that although PD promotes the recovery of islet cell function and insulin secretion, the amount of insulin secreted may be insufficient to effectively improve lipid metabolism disorders. As noted in reference [28], lipid accumulation within pancreatic *β*‐cells (lipotoxicity) is a key factor leading to *β*‐cell dysfunction. The discrepancy between our findings and those of Jin et al. [4], who reported that PD dramatically decreased TC, TG, and LDL‐C, is probably associated with the different experimental designs. The present work employed continuous high‐fat feeding to stably simulate human diabetic metabolic conditions, whereas reference [4] used standard chow for modeling. The more severe insulin resistance and lipid metabolic diseases resulting from the HFD may have attenuated the lipid‐lowering effects of PD.

### 4.1. Limitations

There are several limitations in this work. First, our in vivo experiments were conducted exclusively in animal models, and the results may not be directly translatable to humans because of species differences in metabolism, disease progression, and genetic backgrounds. Future clinical studies should be conducted to validate the efficacy of PD among diabetic patients. Second, although we identified TNF as a critical target and explored its role in the TNF/TNF‐R1/caspase‐3 axis, the interactions of PD with other potential targets and pathways remain to be fully elucidated. Further investigation into a broader range of molecular mechanisms will provide a more comprehensive understanding of the effects of PD on diabetic islet *β*‐apoptosis. Third, the lipid metabolism results were inconsistent with those of some previous reports, highlighting the need for more standardized experimental designs and larger sample sizes to verify the lipid‐regulatory function of PD. Finally, the long‐term efficacy of PD administration on islet activity and metabolic health requires further investigation. Addressing the abovementioned limitations is crucial for advancing our understanding of the therapeutic applications of PD in diabetes management.

## 5. Conclusions

The present work employed network pharmacology, bioinformatics, and experimental methods to elucidate the mechanism by which PD ameliorates pancreatic *β*‐apoptosis in DM. Our findings demonstrate that PD attenuates diabetic *β*‐apoptosis through inhibition of the TNF/TNF‐R1/caspase‐3 signaling axis, thereby providing a potential therapeutic strategy for treating DM.

## Author Contributions

**Jun Li:** conceptualization, data curation, visualization, writing—original draft, funding acquisition, project administration. **Yifan Zhou:** data curation, visualization, writing—original draft. **Xuetong Wu:** visualization, writing—original draft. **Guo Cheng:** data curation, writing—original draft. **Qun Ma:** data curation, funding acquisition. **Yixue Zhou:** data curation. **Nannan Zhang:** conceptualization, data curation, funding acquisition, project administration, supervision, writing—review and editing.

## Funding

This study was supported by the Guizhou Provincial Basic Research Program (General Program) (QKHJC‐MS [2026]671); Guizhou Provincial Department of Education Youth Science and Technology Talents Growth Project (Qianjiaoji [2024] No.116).

## Disclosure

All authors have reviewed and approved the final version of the manuscript and agree to be accountable for all aspects of the work. Corresponding author (Nannan Zhang) had full access to all of the data in this study and takes complete responsibility for the integrity of the data and the accuracy of the data analysis.

## Ethics Statement

This study was reviewed and approved by the Guizhou University of Traditional Chinese Medicine Institutional Animal Care and Use Committee (Approval No.: 20230224). All experimental protocols strictly complied with the ARRIVE guidelines for animal experiments. The study is reported in accordance with ARRIVE guidelines (https://arriveguidelines.org). Min6 cells used in this study were authenticated commercial cell lines purchased from Shanzi Biotechnology (Wuhan, China). No human tissue or patient samples were involved in the research.

## Conflicts of Interest

The authors declare no conflicts of interest.

## Supporting Information

Additional supporting information can be found online in the Supporting Information section.

## Supporting information


**Supporting Information 1** File S1.

**Supporting Information 2** File S2.

## Data Availability

The authors confirm that the data supporting the findings of this study are available within the article and/or its Supporting Information.
