# Supplementary material for: Polydatin Reverses Diabetes‐Induced Apoptosis of Pancreatic β‐Cells by Inhibiting the TNF/TNF‐R1/Caspase‐3 Signaling Axis
Source: J Diabetes Res. 2026 Sep 12;2026:9073132. doi: 10.1155/jdr/9073132 (PMC13570329; doi:10.1155/jdr/9073132)
Supplement: Supplementary file 1 [file JDR-2026-9073132-s001.docx]

Supplementary file 1

**Results**

**Oral glucose tolerance test (OGTT) in mice**

Blood glucose (mg dL⁻¹) was measured at 0, 30, 60, 90, and 120 min after glucose challenge. Compared with controls, the model group displayed sustained hyperglycaemia at every post-dose interval. PD administration—low or high dose—further impaired glucose tolerance, with the high-dose cohort exhibiting the most severe elevation.

**Supplementary Figure 1 Oral glucose tolerance test (OGTT) in mice**

Data are expressed as mean ± SEM; n = 8 mice per group.
