# Supplementary material for: Polydatin Reverses Diabetes‐Induced Apoptosis of Pancreatic β‐Cells by Inhibiting the TNF/TNF‐R1/Caspase‐3 Signaling Axis
Source: J Diabetes Res. 2026 Sep 12;2026:9073132. doi: 10.1155/jdr/9073132 (PMC13570329; doi:10.1155/jdr/9073132)
Supplement: Supplementary file 2 [file JDR-2026-9073132-s002.docx]

Supplementary file 2

**Results**

**Effects of PD on serum lipid profiles in mice**

Serum lipid analyses revealed that, rather than decreasing LDL-C, TG, or TC, PD administration actually elevated the levels of these three parameters. Although HDL-C was also increased by PD, the change did not reach statistical significance.

**Supplementary Figure 2. Effects of PD on serum lipid profiles in mice**

A: LDL-C; B: HDL-C; C: TG; D: TC

Data are presented as mean ± SEM; n = 8 per group.

**P* < 0.05，***P* < 0.01，****P* < 0.001，and *****P* < 0.0001
